# Genomic evidence for ancient human migration routes along South America's Atlantic coast

**DOI:** 10.1098/rspb.2022.1078

**Published:** 2022-11-09

**Authors:** Andre Luiz Campelo dos Santos, Amanda Owings, Henry Socrates Lavalle Sullasi, Omer Gokcumen, Michael DeGiorgio, John Lindo

**Affiliations:** ^1^ Department of Electrical Engineering and Computer Science, Florida Atlantic University, Boca Raton, FL 33431, USA; ^2^ Department of Archaeology, Federal University of Pernambuco, Recife, Pernambuco 50670-901, Brazil; ^3^ Department of Anthropology, Emory University, Atlanta, GA 30322, USA; ^4^ Department of Biological Sciences, State University of New York at Buffalo, Buffalo, NY 14260, USA

**Keywords:** archaeogenomics, ancient migrations, Northeast Brazil, settlement of the Americas

## Abstract

An increasing body of archaeological and genomic evidence has hinted at a complex settlement process of the Americas by humans. This is especially true for South America, where unexpected ancestral signals have raised perplexing scenarios for the early migrations into different regions of the continent. Here, we present ancient human genomes from the archaeologically rich Northeast Brazil and compare them to ancient and present-day genomic data. We find a distinct relationship between ancient genomes from Northeast Brazil, Lagoa Santa, Uruguay and Panama, representing evidence for ancient migration routes along South America's Atlantic coast. To further add to the existing complexity, we also detect greater Denisovan than Neanderthal ancestry in ancient Uruguay and Panama individuals. Moreover, we find a strong Australasian signal in an ancient genome from Panama. This work sheds light on the deep demographic history of eastern South America and presents a starting point for future fine-scale investigations on the regional level.

## 1. Introduction

The Americas were the last continents populated by humans, with an increasing body of archaeological and genomic evidence indicating a complex settlement process starting from Beringia around the Last Glacial Maximum, approximately 20 000 calendar years before present (BP) [1–7]. Recent studies involving both ancient and present-day genomes have described how the ancestral Native Americans (NAs) further explored and settled northern North America and later diverged into two basal branches called Northern NA (NNA or ANC-B) and Southern NA (SNA or ANC-A) [3,4,7–11]. The SNA lineage, represented by the Clovis-associated Anzick-1 and the Spirit Cave individuals, is an ancestral component in present-day Central and South Americans, indicating that multiple groups related to this branch crossed Mesoamerica and entered South America. An additional nuanced ancestry to South America [1,7,8,10,11] may derive from an unsampled population (termed ‘Ypikuéra population’ or ‘Population Y’), which may have contributed to the early peopling of South America by introducing an Australasian shared ancestry that is observed in contemporary Indigenous Amazonian groups (e.g. Surui and Karitiana) [1,6,9,12,13]. To date, only one ancient individual (Sumidouro5, also one of the oldest representatives of the SNA lineage), unearthed in the Lagoa Santa archaeological area in Southeast Brazil, has been found to harbour the Australasian signal [7,9]. Vast portions of the southern continent, however, remain largely unexplored by archaeogenomic studies.

One such area is Northeast Brazil, along the Atlantic coast. Northeast Brazil houses some of the richest archaeological sites in South America [14–16] but has yielded only a single low-coverage ancient human genome to date (Enoque65, from Serra da Capivara archaeological area) [5] (electronic supplementary material). In the light of Brazil's geographical extension, the archaeogenomic study of its Northeast Region may reveal important demographic aspects underlying many of the events that composed the settlement of South America, including putative migratory movements from North and Central America—through the Southern Cone along the Atlantic coast. The study of these still poorly characterized events from a genomic perspective, especially at the regional level, may lead to the disclosing of key chapters of the demographic history of the Americas [1,3,8,9].

**Figure 1.**
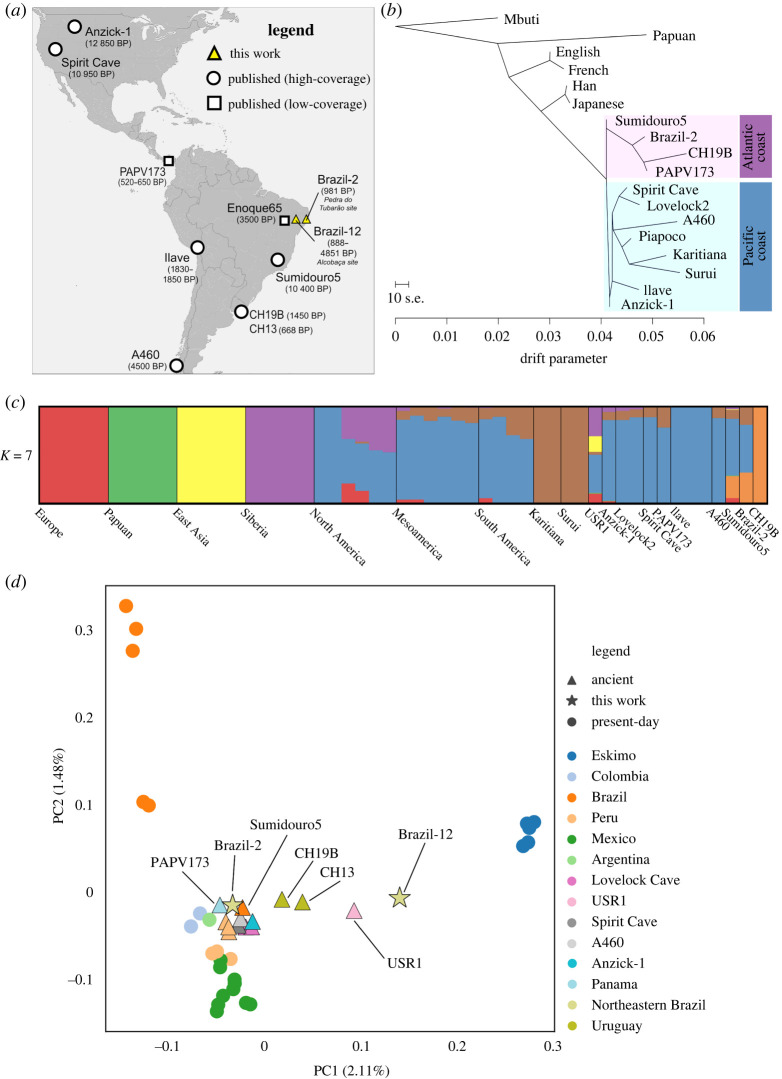
Ancient samples overview and broad genomic affinity. (*a*) Location of the newly sequenced ancient Brazilian samples and previously published ancient individuals. Ilave corresponds to individuals IL2, IL3 and IL7 from Peru. The chronological range associated with Brazil-12 represents contextual data (electronic supplementary material). (*b*) TreeMix maximum likelihood tree showing the distinct Pacific and Atlantic clades highlighted. Only the ancient samples from Brazil and Uruguay with the highest sequencing depth were used. (*c*) ADMIXTURE analysis for *K* = 7 clusters. Bars represent individuals, whereas each colour represents a distinct ancestral component, with bar height representing the proportion of that component comprising a given individual. See electronic supplementary material, figure S1 for additional plots assuming different values for *K*. (*d*) Principal component analysis (PCA) on present-day individuals with ancient individuals projected onto the PCs 1 and 2. See electronic supplementary material, figure S2 for additional PCA plots. Percentages along axis labels correspond to proportions of variance explained by PCs. (Online version in colour.)

Here, we report newly sequenced genomes from two ancient human individuals (Brazil-2 and Brazil-12) unearthed in two different archaeological sites in Northeast Brazil: Pedra do Tubarão and Alcobaça (figure 1*a*). Both archaeological sites are located in the state of Pernambuco and are associated with the Agreste rock art tradition, the second most representative rock art tradition in Northeast Brazil. It is believed that this tradition emerged at Serra da Capivara archaeological area, state of Piauí, approximately 5000 years BP and later dispersed to other portions of Northeast Brazil. In Pernambuco, the oldest dates associated with this tradition go as far as 2000 years BP. Brazilian archaeologists in Northeast Brazil have pointed to the challenges of affiliating rock art traditions and other material records at the archaeological sites in the area [17]. Thus, the chronological boundaries of putatively Agreste-affiliated archaeological cultures are still not precisely defined. There is also no record of post-European-contact Indigenous occupation of these sites, indicating a loss of cultural continuity in the area (electronic supplementary material).

The sequencing achieved a mean depth of approximately 10× for Brazil-2 and 8× for Brazil-12. Based on mitochondrial DNA, we estimated modern human contamination to be around 3.6% for Brazil-2 and 5% for Brazil-12, and mitochondrial haplogroups C1b and D1a2, respectively. Brazil-2 has also been directly dated to approximately 981 years BP (electronic supplementary material), with its molecular sex estimated to XY, belonging to Y-chromosomal haplogroup Q1b1a1a1 (table 1). Along with the Northeast Brazil samples, we further investigated two recently sequenced ancient genomes from Uruguay (CH13 and CH19B) [20] (figure 1*a*). Our aim is to characterize, at the regional level, dispersal and admixture events involving the ancient individuals and populations of the Americas, particularly along South America's Atlantic coast.

**Table 1.** Ancient individuals sequenced in this study and associated information. Radiocarbon dating of Brazil-2 was conducted at the University of Arizona AMS Laboratory. Therefore, the upper and lower boundaries presented here are, respectively, the mean values of the youngest and oldest ages obtained in the site (electronic supplementary material). Y-chromosomal and mitochondrial haplogroups were estimated using Y-SNP Haplogroup Hierarchy Finder [18] and Haplocheck [19], respectively.

sample	site	^14^C age (uncal. BP)	mitochondrial haplogroup	Y-chromosomal haplogroup	contamination (%)	endogenous DNA (%)	mean read depth	genetic sex
Brazil-2	Pedra do Tubarão, Brazil	981 ± 23	C1b	Q1b1a1a1	3.6	11.65	10.67	XY
Brazil-12	Alcobaça, Brazil	888 ± 25–4851 ± 30^a^	D1a2	N/A	5.0	3.41	8.32	not assigned

^a^Based on 24 traditional radiocarbon dates obtained from charcoals unearthed in the Alcobaça site.

## 2. Results

### (a) A distinct genomic relationship between ancient Brazil, Panama and Uruguay

We investigated the ancient individuals' broad genomic relationships to other populations using maximum likelihood trees [21], model-based clustering [22] and principal component analysis (PCA) [23] (figure 1*b–d*) (Material and methods). We first evaluated the evolutionary relationship among ancient NAs (aside from the above-mentioned ancient genomes we also included the Andeans IL2, IL3 and IL7 from the Ilave region in Peru [24], the Chilean A460 [9] and PAPV173 from Panama [1]) and present-day worldwide populations via TreeMix [21]. The analysis separated the ancient NAs into two distinct clades (figure 1*b*). The first is composed of previously published samples unearthed near the Pacific coast of the Americas [9,24,25], in addition to the present-day Piapoco, Surui and Karitiana from the Amazonian rainforest [26]. The second clade comprises Sumidouro5 [9] and Brazil-2 from Brazil, CH19B [20] from Uruguay and PAPV173 from Panama [1]. While the first mentioned clade is representative of the ancient Americas’ Pacific coast, the second is composed of ancient individuals unearthed in archaeological sites closer to South America's Atlantic coast, though we acknowledge that PAPV173 was found along Panama's Pacific coast [1]. Still on the Atlantic clade, the resulting maximum likelihood tree indicates that Sumidouro5 is a possible ancestor of Brazil-2 (as it has no estimated genetic drift since diverging with Brazil-2), which in turn is associated with an ancestral branch of PAPV173 (and CH19B), a finding that suggests a south-to-north directionality. This result is consistent with the associated chronological data (i.e. Sumidouro5 is older than Brazil-2, which is more ancient than PAPV173). On the other hand, the Pacific clade appears to summarize the body of knowledge [7] around the settlement of the Americas, in a north-to-south directionality.

We used ADMIXTURE to explore the genomic structure of the ancient NAs in the context of a worldwide reference panel [22]. When assuming *K* = 7 clusters, chosen based on the lowest cross-validation value, we found that Brazil-2, CH19B and Sumidouro5 share proportions of a distinct component, represented by the orange colour. CH19B's structure, specifically, is totally made up of the orange component. This ancestral component is restricted to ancient individuals unearthed closer to South America's Atlantic coast. Interestingly, Sumidouro5 harbours a barely notable proportion of the green component, only found in present-day Papuans and in a few ancient samples from North America (USR1 and Anzick-1, also barely notable) (figure 1*c*; electronic supplementary material, figure S1).

Similarly, the PCA results show that Sumidouro5 falls between Brazil-2 and CH19B along PC1, with PAPV173 positioned very close to Brazil-2 (figure 1*d*; electronic supplementary material, figure S2). With the exception of the USR1 individual from Alaska, all other previously published ancient NAs are tightly clustered in proximity to Brazil-2, Sumidouro5 and PAPV173. This ancient cluster almost overlaps with present-day Peruvians and Argentinians, while present-day individuals from Mexico and Colombia are also in close proximity. At first glance, the clustering positions imply that Brazil-2 is more closely related to other present-day NAs than to the Karitiana and Surui from the Brazilian Amazonia. However, the observed distance between the ancient and present-day Brazilian samples is likely the result of strong genetic drift effects experienced by the Amazonian populations [6,12] (figure 1*b*) since they split with the ancient individuals. It is known that changes in allele frequencies due to drift in a given population might affect its position in PC-space [27] relative to other populations. The exception to this observation is when the referred population is not part of the principal components' (PCs) construction [27], which is not the case for Surui and Karitiana. In this context, outgroup *f*_3_ statistics (results provided in the *Surui and Karitiana harbour the highest affinity with ancient Americas* subsection further ahead) are a better suited [28] technique to assess the genomic affinity between the ancient and present-day Brazilian individuals. Brazil-12, on the other hand, falls closer to present-day Eskimo individuals than to any other present-day or ancient NAs, whereas CH13 (Uruguay) falls in the vicinity of USR1. It is important to note here that Brazil-12 and CH13 are shallow genomes compared to the other ancient individuals, which may explain the more distant clustering positions within the PCA plot. When PCA is performed on the same dataset as the ADMIXTURE analysis (figure 1*c*), we find that the placement of the ancient and present-day samples of the Americas are almost identical as in the original PCA results (figure 1*d*; electronic supplementary material, figure S2). However, CH19B now clusters with Siberians and USR1 when PC1 and PC2 are considered (electronic supplementary material, figure S3*a*), while also falling closer to the Cree regardless of the pair of PCs visualized (electronic supplementary material, figure S3).

### (b) Deep archaic and Australasian ancestries in South America and Panama

**Figure 2.**
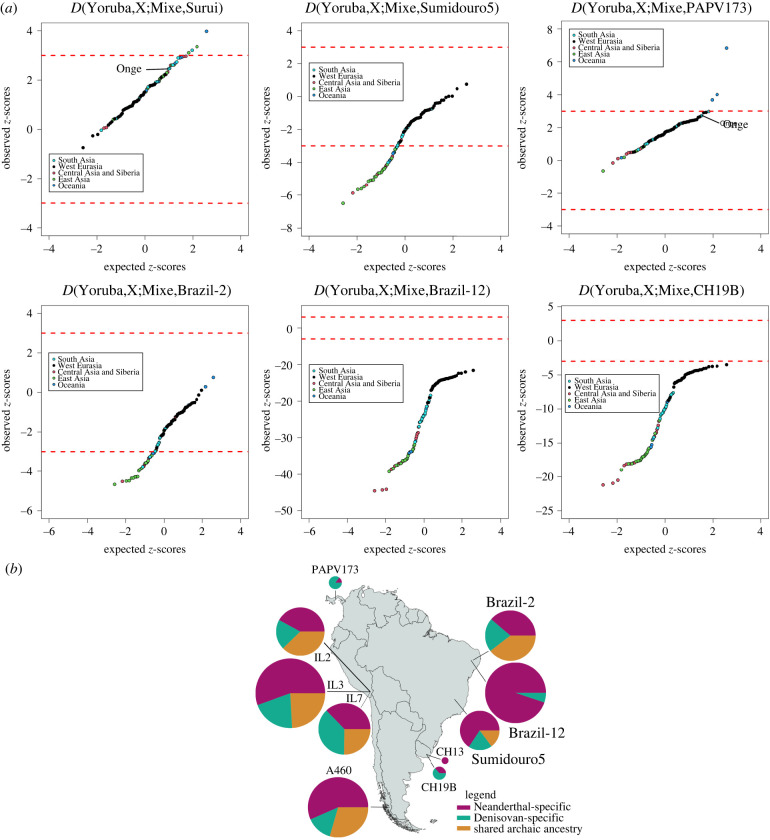
Deep ancestries of the ancient individuals of the Americas. (*a*) Quantile–quantile plots of the *Z*-scores for the *D*-statistic test for each ancient sample, a non-American, non-African population ‘X’ from Simons Genome Diversity Project (SGDP) [26] and the Mixe [26], compared to the expected ranked quantiles for the same number of normally distributed values. Each data point represents a distinct population from SGDP. Dashed red lines represent significance thresholds. (*b*) Archaic ancestry in ancient South America and Panama. Pie chart radius reflects the proportion of shared archaic loci in the individual. (Online version in colour.)

We further explored the ancient individuals’ deep genomic ancestries using *D*-statistics [29] and identity by descent (IBD) analysis [30] (figure 2) (Material and methods). We first evaluated the presence of the Australasian signal along South America's Atlantic coast computing *D*-statistics of the form *D*(Yoruba,X;Mixe,TestPop), where X is a present-day non-African population and where TestPop is set as either Surui, Sumidouro5, PAPV173, Brazil-2, Brazil-12, CH19B, CH13 or Anzick-1. A similar *D*-statistics analysis was previously used to report this signal in the ancient Lagoa Santa genome (Sumidouro5) [9] and present-day Surui [6]. While we were able to replicate the Australasian signal in the Surui, we do not find the signal in Sumidouro5 as previously reported [9], possibly due to different reference panels being used (figure 2*a*). The signal is also not present in Brazil-2, Brazil-12, CH19B, CH13 and Anzick-1 (figure 2*a*; electronic supplementary material, figure S4). We do find, however, that Papuans, New Guineans and Indigenous Australians share significantly more alleles with PAPV173 than with the Mixe (*Z* > 3). Moreover, in the instances in which the Surui and PAPV173 were compared to the Mixe in relation to the Onge, a previously reported ‘attenuated signal’ [6] can be found (*Z* ≈ 2.7) (figure 2*a*). To corroborate these results, we tested some of the ancient samples using *D*-statistics of the form *D*(Yoruba,TestPop;X,B), in which B is set either as English, Han, Mixe, Papuan or Surui. Using this new form, we find that Sumidouro5 shares significantly more alleles with the Andamanese Onge (*Z* < −3) when B is the Papuans, which can represent the previously reported Australasian signal in this ancient sample [9] (electronic supplementary material, table S1).

To investigate even deeper genomic ancestry, we used IBDmix [30] to test all South American ancient individuals highlighted in this work (and the Panamanian PAPV173) for the presence of putative archaic (Altai Neanderthal [31] and Denisovan [32]) genomic contributions. We found that all samples share a very small genomic proportion with at least one of the archaic human species used as a reference (figure 2*b*). Interestingly, PAPV173 and CH19B harbour greater Denisovan- than Neanderthal-specific ancestry. When performing cluster analysis based only on the archaic proportions, these two samples cluster together (electronic supplementary material, figure S5), despite being situated more than 5000 km and almost 1000 years apart, and is consistent with previous findings [20].

To corroborate the IBDmix results, we ran several *f*_4_-ratio tests to detect Denisovan-related ancestry in the high-coverage ancient samples: Brazil-2, IL2, IL3, IL7, Sumidouro5 and A460. We restricted our tests to the regions found to harbour archaic ancestry in the IBDmix analysis (i.e. the IBD tracks). All the non-African populations from Simons Genome Diversity Project (SGDP) public dataset were organized into super-/continental populations (Americas, Central Asia/Siberia, East Asia, South Asia and West Eurasia) and used here as baselines, i.e. we compare the proportion of Denisovan ancestry in the ancient individuals with the proportion harboured by the present-day superpopulations, one at a time (electronic supplementary material). The resulting statistic (*α*) is defined as the ratio between two *f*_4_-statistics [33], and we use an already established *f*_4_-ratio form to test for the Denisovan-related ancestry [34,35] (electronic supplementary material). Regardless of the baseline used, we find a positive correlation between the *f*_4_-ratio *α* and the proportion of Denisovan-related ancestry among the total archaic ancestry identified by IBDmix (electronic supplementary material, figure S6 and table S2). We recognize, however, that due to the small number of ancient samples tested here our results do not attain statistical significance.

### (c) Surui and Karitiana harbour the highest affinity with ancient Americas

**Figure 3.**
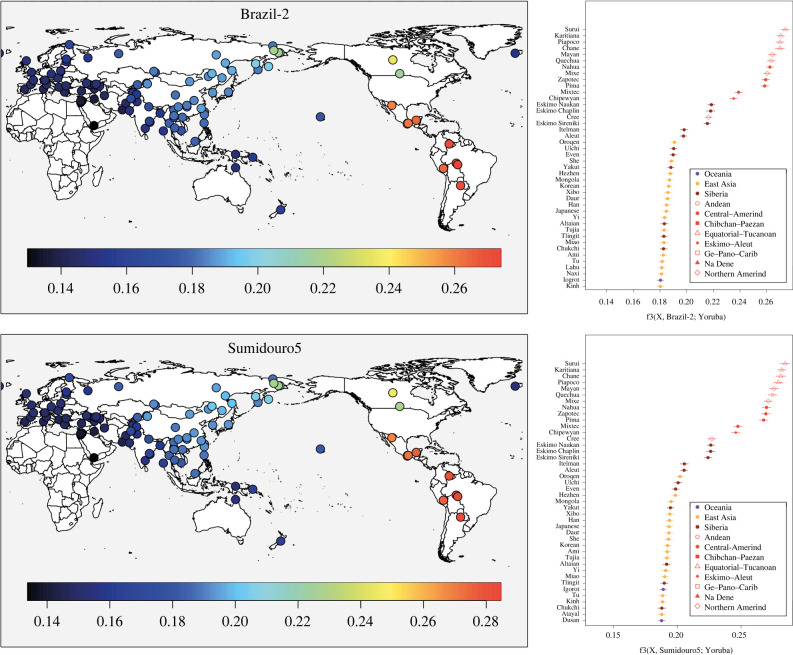
Top genomic affinities of ancient genomes from Brazil. Heatmaps and ranked results of outgroup *f*_3_-statistics analyses showing that Surui and Karitiana harbour the highest affinities with Brazil-2 and Sumidouro5, contrarily to what PCA implied (figure 1*d*). (Online version in colour.)

We used outgroup *f*_3_ statistics [33] to further highlight the shared genomic history between the ancient individuals and present-day populations (figure 3). Contrarily to what the PCA results implied (figure 1*d*; electronic supplementary material, figure S2), ranked outgroup *f*_3_ analyses demonstrate that Brazil2, Sumidouro5, PAPV173, CH19B, CH13 and Spirit Cave are more genomically related to Surui and Karitiana than to any other present-day population (figure 3; electronic supplementary material, figure S7).

### (d) Dispersal in South America led to eastern two-way migration route

**Figure 4.**
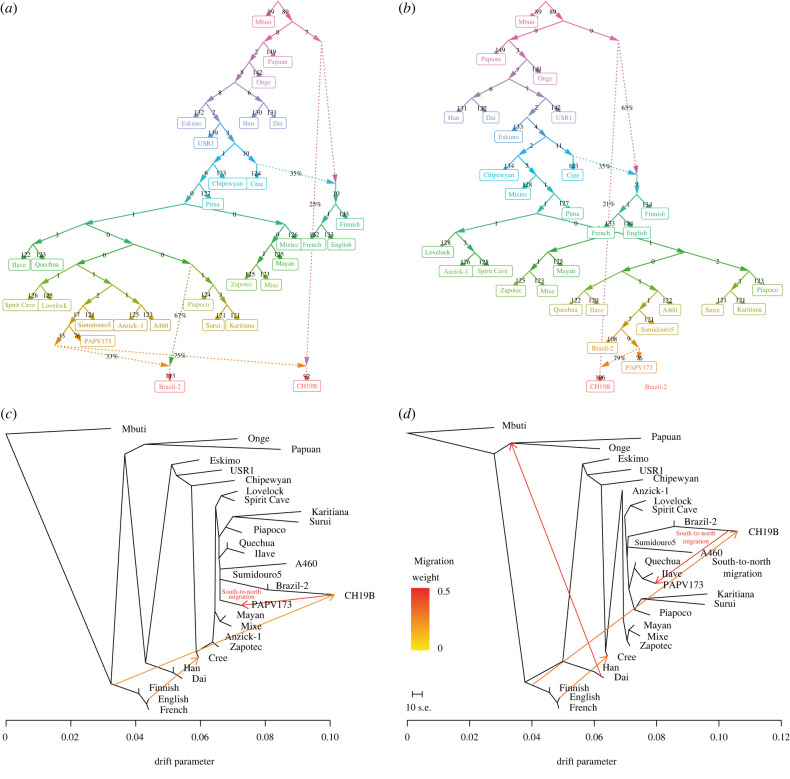
Demographic history of the ancient populations of South America. (*a*) and (*b*) Demographic models estimated by qpGraph with three and two migration events, respectively, showing populations splits in South America and the relationship between A460, Sumidouro5, Brazil-2, PAPV173 and CH19B. (*c*) and (*d*), Demographic models estimated by TreeMix with three and four migration events, respectively, showing south-to-north migration events linking CH19B and PAPV173. Alternative TreeMix graphs with one and two migration events are provided in electronic supplementary material, figure S8. (Online version in colour.)

Lastly, we explored the demographic history of the ancient South American individuals by using demographic modelling information [33]. We used qpGraph [33] to build demographic models involving a reference panel of selected present-day worldwide populations and almost all the previously mentioned ancient individuals of the Americas (with the exceptions of Brazil-12 and CH13). The topology of the best-fit model, with three migration events, shows that population splits occurred after the first human groups reached South America's western/Andean portion (as indicated by Quechua's and Ilave's position) (figure 4*a*). Brazil-2's ancestry can be traced back both to a clade formed by Sumidouro5 and PAPV173, and to an ancestral branch of the present-day Piapoco, Surui and Karitiana. CH19B also received a big genomic contribution stemming from the clade formed by Sumidouro5 and PAPV173, while still inheriting genomic contribution from a possibly unsampled basal population, as previously reported [20] (figure 4*a*). Interestingly, the graph with two migration events shows that A460, Sumidouro5, Brazil-2 and PAPV173 form a clade by themselves, with CH19B receiving a large contribution from this clade, which is similar to the TreeMix result in figure 1*b*. This model suggests that the settlement of the Atlantic coast occurred only after the peopling of most of the Pacific coast (and the Andes). The Piapoco, Surui and Karitiana again form a distinct clade (figure 4*b*).

Similarly, when maximum likelihood graphs involving the same samples/populations (and three and four migration events) are estimated using TreeMix, it is possible to observe Brazil-2, Sumidouro5 and CH19B forming a distinct clade, with PAPV173 and A460 as the nearest branches. These samples diverge only after the branching of an Andean clade formed by the Quechua and the Ilave ancient samples. Moreover, both results show a south-to-north migration event linking CH19B and PAPV173, which appears to be analogous to the genomic contributions originating in the branch of PAPV173 in both qpGraph results with two and three migration events (figure 4*a*,*b*). Moreover, another migration event estimated by TreeMix stems from the English and heads toward the Cree (figure 4*c*,*d*), which is similar to a genomic contribution observed in the qpGraph results (figure 4*a*,*b*) that originates in the Cree and heads toward the European clade composed of Finnish, English and French populations. A third migration event estimated in the TreeMix analysis (from the Dai to the ancestors of the Onge and Papuans; figure 4*d*) finds no correspondent contribution in the qpGraph results (figure 4*a*,*b*). Last, we see a basal migration event heading to CH19B, similar to the basal genomic contribution shown in the qpGraph results (figure 4*a*,*b*).

## 3. Discussion

**Figure 5.**
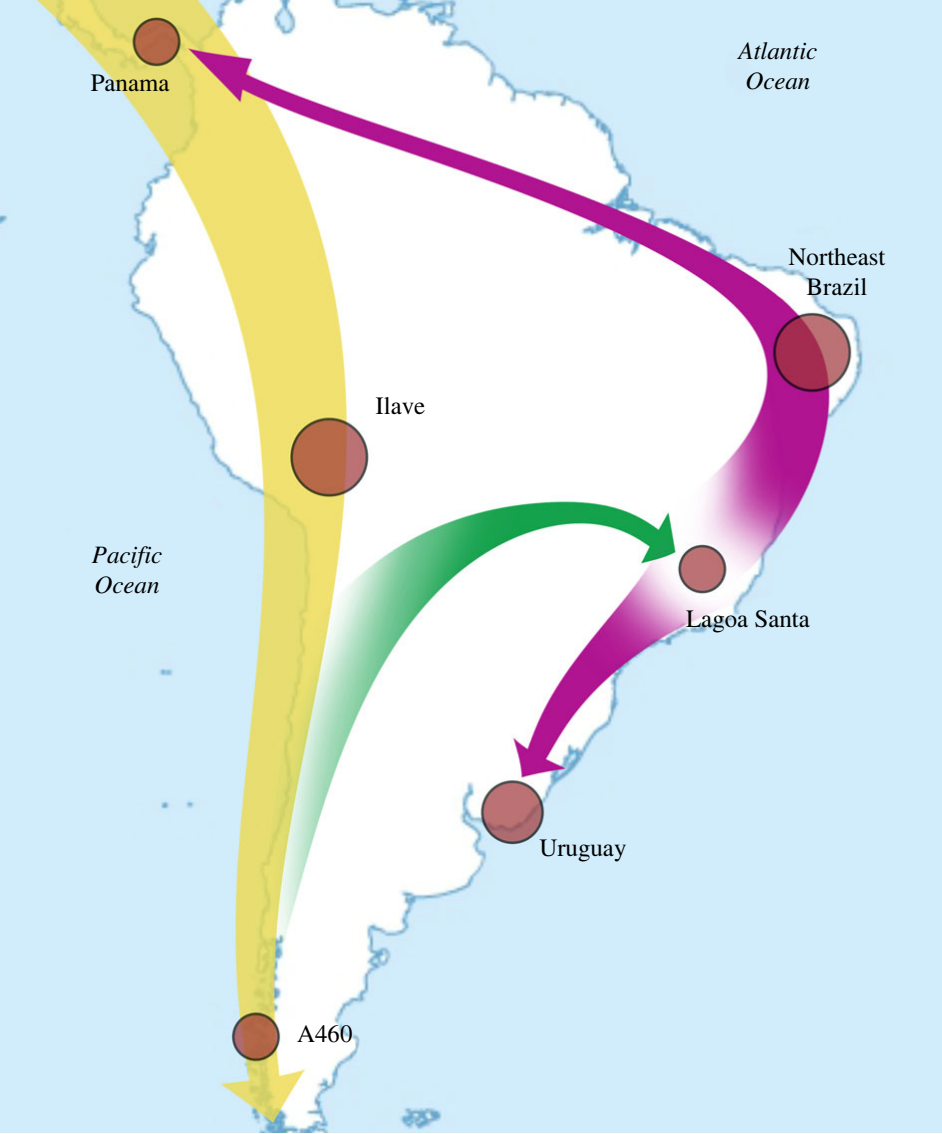
Hypothesis for ancient migrations in South America. The first SNA groups entered South America and spread through the Pacific coast settling the Andes (yellow arrow). At least one population split occurred soon after, branching the first groups that settled the Atlantic coast (green arrow) from the groups that gave rise to the ancient populations of Southern Cone. New migrations may have then emerged along the Atlantic coast, with a possible origin around Lagoa Santa, heading north toward Northeast Brazil and Panama, and south to Uruguay. Eventually, Uruguay and Panama were linked by a south-to-north migration route closer to the Atlantic coast (purple double-headed arrow). (Online version in colour.)

Consistent with previously reported data [1], our results suggest that at least one population split probably occurred not long after the first SNA groups reached the southern portion of the Americas (figures 1*b* and 4*a*,*b*). Based on the qpGraph results, we can hypothesize that this split took place around the Andes, later giving rise to ancient Southern Cone populations and the first groups that settled the Atlantic coast (figures 4*a*,*b* and 5). In light of Sumidouro5's associated chronology—the oldest South American analysed here—it is possible to affirm that the split occurred at least 10 000 years ago. Because Sumidouro5 is associated with the ancestors of both Brazil-2 and CH19B (figures 1*b* and 4), we can further conjecture that new migrations may have then emerged along the Atlantic coast, with Lagoa Santa as the putative geographical source of waves that headed in north-to-south and south-to-north directions—with the latter seemingly reaching Panama (figures 1*b*, 4*a*,*b* and 5). We conclude this hypothesis proposing that human movements closer to the Atlantic coast eventually linked Uruguay and Panama in a south-to-north migration route (figures 4*c*,*d* and 5). The migrations along the Atlantic coast apparently left no trace in the populations closer to the Pacific, as we could not find back-migration events in that direction (figure 4*c*,*d*).

Overall, our results show a strong genomic relationship among Brazil-2, CH19B, Sumidouro5 and PAPV173 (figures 1*b*–*d* and 4). Apart from the occurrence of mass burials in the sites that yielded these samples, there is no other evidence in the archaeological record that indicate shared cultural features between them. It is also important to note that Sumidouro5 is approximately 9000 years older than the other three mentioned individuals, enough time for expected and notable cultural divergence. Moreover, Brazil-2, CH19B and PAPV173, though more similar in age, were located thousands of kilometres apart from each other. Therefore, cultural differentiation is also expected among them [36]. On the other hand, our results further corroborate previously reported evolutionary relationships between PAPV173 and CH19B [20] by providing evidence of a distinct genomic affinity involving archaic human ancestry (figure 2*b*; electronic supplementary material, figure S5).

A strong signal of Australasian ancestry, previously reported only for the Lagoa Santa individual [9] and present-day Surui [6], was also observed for the previously published PAPV173, from Panama [1] (figure 2*a*). The Piapoco, Surui and Karitiana, however, harbour high affinities with Brazil-2 (figures 3 and 4*a*), and thus may have received contributions coming from Central America (in the form of the Australasian signal, in a north-to-south directionality) and South America's Atlantic coast (in a south-to-north directionality).

Together, these results represent substantial genomic evidence for ancient migration events along South Americas' Atlantic coast. Moreover, these events seem to have occurred as an outcome of the migratory waves that originated the first South American populations near the Pacific coast. With these findings, we contribute to the unravelling of the deep demographic history of South America at the regional level.

## 4. Methods

### (a) Experimental design

Northeast Brazil harbours some of the richest and most diverse archaeological areas in the Americas [17], yet it remains largely unexplored by archaeogenomic studies. In light of Brazil's geographical extension and position, genomic data of ancient individuals from the Northeast Region may reveal important aspects underlying many of the events that composed the settlement of South America along the Atlantic coast. We thus extracted and sequenced DNA from two archaeological individuals unearthed in Northeast Brazil to investigate ancient demographic and evolutionary aspects of the region. For this study, we employed established extraction and sequencing protocols (electronic supplementary material), along with a variety of bioinformatics tools and statistical methods, such as: TreeMix [21], ADMIXTURE [22], PCA [23], *D*-statistics [6,29], IBDmix [30], *f*_4_-ratio [33], Outgroup *f*_3_ [33] and qpGraph [33].

### (b) Reference datasets

In this study, the genomic data of 13 ancient human individuals from the Americas were used as a comparative dataset for the analyses of the Northeast Brazil samples. These 13 ancient samples from the Americas were selected based on two criteria: mean sequence depth and geographical sampling location. Our goal was to gather comparative data from the most diverse regions within the Americas while still striving for the highest-quality genomes available for a given geographical region. Thus, we chose USR1 from Alaska [4] as the representative of ancient Beringia; Anzick-1 from Montana, and three samples (Spirit Cave, Lovelock 2 and Lovelock 3) from the Spirit and Lovelock caves in Nevada to represent the Southern North American (SNA) branch [9,25]; PAPV173 from Panama for Central America [1]; IL2, IL3 and IL7 for the Andes in Peru [24]; A460 from the western Southern Cone (Chile) [9]; CH19B and CH13 from the eastern Southern Cone (Uruguay) [20] and Sumidouro5 from the Lagoa Santa archaeological area in Brazil [9], the geographically closest sample to Northeast Brazil (figure 1*a*; electronic supplementary material, data S1). The ancient comparative dataset was complemented with two archaic human samples, Altai Neanderthal [31] and Denisova [32], both from the Denisova Cave in Russia. These two archaic samples represent the highest-quality genomes available, in terms of sequence read depth, for Neanderthals and Denisovans, respectively, and were used to assay archaic ancestry in the ancient samples. Finally, we also employed a reference panel of 253 present-day individuals from the SGDP [26]. This present-day dataset was specifically chosen because it is composed of worldwide Indigenous populations assumed to span much of the human genomic variation [26].

### (c) Treemix analysis

We started with the filtered ancient dataset of called genotypes with transitions removed (electronic supplementary material). TreeMix [21] was applied on the dataset to generate maximum likelihood trees and admixture graphs from allele frequency data. The Mbuti from the Simons dataset [26] was used to root the tree (with the ‘–root’ option). We accounted for linkage disequilibrium by grouping *M* adjacent sites (with the ‘–k’ option), and we chose *M* such that a dataset with *L* sites will have approximately *L*/*M* ≈ 20 000 independent sites. At the end of the analysis (i.e. number of migrations), we performed a global rearrangement (with the ‘–global’ option). We performed 20 iterations for each admixture scenario, choosing the highest likelihood for each. We considered admixture scenarios with m∈{0,1,2,3,4} migration events. The log-likelihood values for the topologies were −1360.4808, 166.6917, 708.4328, 1049.8742 and 1526.6028 for zero to four migration events, respectively. A total of 726 182 overlapping sites were used. The analysis no with migrations (*m* = 0) was also performed with a dataset presenting fewer present-day populations, but the same number of overlapping sites. This was mainly done for better figure readability and resolution, and this result is presented in figure 1*b*.

### (d) ADMIXTURE analysis

We used the ADMIXTURE v.1.3 software to explore the genomic ancestral components in our dataset. The program computes a matrix of ancestral components proportions for each individual (*Q*) and provides a maximum likelihood estimate of allele frequencies for each ancestral component (*P*) [22]. Our dataset was investigated by specifying various numbers of hypothetical ancestral components (*K*). We ran ADMIXTURE assuming values from *K* = 2 to *K* = 8. The best run (i.e. the optimal value for *K*, with the most likely ancestral proportions) was selected based on the lowest 10-fold cross-validation error after 100 analysis iterations, by using the ‘–*cv* = 10’ and ‘-C 100’ flags, respectively. After pruning the dataset for LD, 123 151 overlapping sites were used.

### (e) Principal component analysis

PCA was performed using the ‘smartpca’ program from the EIGENSOFT v.7.2.1 package [23]. The dataset used in this analysis integrated all 12 ancient genomes presented in figure 1*a* plus USR1 [4] and Lovelock Cave [9] individuals, all with transitions removed, and 26 present-day individuals from the SGDP [26] (Chane from Argentina; Karitiana and Surui from Brazil; Piapoco from Colombia; Mayan; Mixe, Mixtec, Prima and Zapotec from Mexico; Quechua from Peru; and Eskimos Chaplin, Naukan and Sireniki from Russia). PCs were calculated using the present-day populations with the ‘poplistname’ and ‘autoshrink: YES’ options. Ancient data, characterized by a large portion of missing sites, were then projected onto the computed PCs with the ‘lsqproject: YES’ option. No outliers were excluded for this analysis, which was based on 2 727 376 loci presenting a genotyping rate of at least 90% across the whole dataset. The PCA with the same dataset as in the ADMIXTURE analysis, with 19 additional present-day individuals from the Cree, Chipewyan, Papuan, Dai, Han, English, French and Finnish populations of the SGDP (electronic supplementary material, figure S3), was based on 2 919 158 SNPs that passed the aforementioned genotyping rate threshold.

### (f) *D*-statistics

The assessment of the Australasian signal in the ancient samples of the Americas and present-day Surui was performed using the POPSTATS Python program [6]. Two forms were used in this analysis. In the first analysis, we ran in the form of *D*(Yoruba,X;Mixe,TestPop), previously used to report the Australasian signal [6,9], in which ‘X’ was all non-African and non-American populations in the Simons Genome Diversity Project [26], while ‘TestPop’ was Brazil-2, Brazil-12, CH13 [20], CH19B [20], Surui [26], Sumidouro5 [9], Anzick-1 [25] or PAPV173 [1]. The analysis and form used were originally proposed to test for a symmetric relationship between non-American populations and Central and Southern NAs, after it was found that genomic variation from populations of Central and South America were inconsistent with the hypothesis of a single ancestral population [6]. These inconsistencies were driven by asymmetric affinities between the Amazonian Surui and Karitiana from South America and the Mixe and Pima from Central America in relation to South Asians and Oceanians [6]. Thus, for this analysis, we selected the Mixe as the representative population of Central America, similar to previous studies [6,9]. In the second analysis, we ran in the form of *D*(Yoruba,TestPop;X,B), in which ‘B’ was the English, Han, Mixe, Papuan and Surui populations from Simons Genome Diversity Project [26], ‘X’ was all the non-African populations from the same project [26] and ‘TestPop’ comprised the same samples used in the previous run, except for Anzick-1 [25] (electronic supplementary material). The aim of this second analysis is to test for a symmetric relationship between present-day non-Africans and ancient NAs. The dataset had all transitions removed. No pruning for linkage disequilibrium was applied. The number of polymorphic sites used in this analysis depends on the coverage of the four populations that are being compared. The minimum number of sites analysed was 143 014 sites in *D* (Yoruba, CH13; Chane, Surui) and the maximum was 3 831 654 sites in *D* (Yoruba, Surui; Palestinian, Papuan).

### (g) Identity by descent analysis

We assessed human archaic ancestry in the ancient samples of South America and Panama using the IBDmix software [30]. The program is able to identify introgressed human sequences using a pair of }‘archaic sample’}-{‘test population’} [30]. Since IBDmix needs at least 10 samples forming a single ‘test population’ to make robust inferences [30], we considered Brazil-2, Brazil-12, Sumidouro5 [9], CH13 [20], CH19B [20], A460 [9], IL2 [24], IL3 [24], IL7 [24] and PAPV173 [1] as a single population (Ancient NAs). This ‘Ancient NAs’ dataset consisted of 5 010 609 polymorphic sites that were kept after transitions removal. No other filtering step was performed for this analysis. IBDmix was then run for each pair {Altai Neanderthal [31] or Denisova [32]}-{‘Ancient NAs’}. A summary of introgressed segments was generated and segments with a LOD score less than 4 were filtered out with the ‘summary_lod: 4’ option. Introgressed segments with length less than 50 kb were also removed with the option ‘summary_length: 50 000’. These are the same thresholds used in IBDmix's original publication [30]. All the other parameters were run on default settings.

### (h) *f*_4_-ratio analysis

We performed *f*_4_-ratio tests to estimate Denisovan-related ancestry in the high-coverage ancient samples of South America using ADMIXTOOLS [33]. The genomes of Altai Neanderthal [31], Denisova [32] and all non-African (with the exception of Yoruba, which are used as an outgroup) populations in the SGDP [26] public dataset were compared using a previously established form [34,35] (electronic supplementary material). The SGDP populations were then organized into superpopulations (Americas, Central Asia/Siberia, East Asia, South Asia and West Eurasia) and used as baselines, i.e. references for the amount of Denisovan-related ancestry [35]. The dataset had all transitions removed, and no pruning for linkage disequilibrium was applied. Only the genomic regions harbouring archaic ancestry according to the IBDmix results were used. The number of polymorphic sites used in this analysis also depends on the coverage of the samples within the five populations that are being used in each test. The minimum number of sites analysed was 1630 when Sumidouro5 was tested, regardless of the baseline, whereas the maximum was 2920 sites when IL3 was tested with the American superpopulation as baseline.

### (i) Outgroup *f*_3_ analysis

We extracted all non-African populations from the Simons Genome Diversity Project [26] as well as the sub-Saharan African Yoruba population to create a reference set of present-day human populations. For a given ancient sample (Sumidouro5, Brazil-2, CH19B, CH13, PAPV173, Anzick-1 or Spirit Cave), we merged variant calls from the ancient sample with the present-day human reference set. We then filtered for SNPs with exactly two distinct alleles observed in the merged set. To compute outgroup *f*_3_ statistics of the form *f*_3_(Present-day, Ancient; Yoruba) where the Yoruba population was considered the outgroup to the Present-day and Ancient human references, we applied the qp3Pop module of ADMIXTOOLS [33]. Because the Sumidouro5, Anzick-1 and Spirit Cave samples were not treated with uracil-DNA glycosylase, we also removed C/T and G/A SNPs to guard against a form of DNA damage. The number of polymorphic sites used in this analysis depended on the triple of populations being compared as well as whether C/T and G/A SNPs were removed from the samples. For the Sumidouro5, Anzick-1 and Spirit Cave samples in which we removed C/T and G/A SNPs, we, respectively, employed a minimum of 768 872, 763 566 and 778 916 sites and a maximum of 837 075, 829 379 and 848 782 sites. For the Brazil-2, CH19B, CH13 and PAPV173 samples, we, respectively, employed a minimum of 2 195 333, 737 689, 284 355 and 875 830 sites and a maximum of 2 363 461, 776 333, 297 652 and 932 752 sites.

### (j) qpgraph

We extracted a subset of individuals from the Simons Genome Diversity Project [26] to create a small global reference panel to explore relationships between ancient (Brazil-2, Sumidouro5, PAPV173, CH19B, USR1, Anzick-1, Spirit Cave, Lovelock, IL2, IL3, IL7 and A460) and present-day samples using an admixture graph. The present-day populations we extracted were from Africa (Mbuti), Europe (Finnish, French and English), Oceania (Papuan), South Asia (Onge), East Asian (Han and Dai), Siberia (Eskimo Chaplin, Eskimo Naukan and Eskimo Sireniki that we jointly refer to as Eskimo in our analyses), North America (Cree, Chipewyan, Pima, Mixe, Mixtec and Zapotec), Central America (Mayan) and South America (Quechua, Piapoco, Karitiana and Surui). We merged this present-day reference panel with variant calls of the twelve ancients. We then filtered for SNPs with exactly two distinct alleles observed in the merged set, removed SNPs with any missing data, and removed C/T and G/A SNPs to guard against DNA damage, resulting in a dataset containing 110 505 SNPs. We applied the R package ADMIXTOOLS2 (https://uqrmaie1.github.io/admixtools/index.html, ADMIXTOOLS2 is currently under preparation) to perform qpGraph [33] estimation. Using this software, we precomputed *f*_2_ statistics between population pairs in a two megabase SNP block. Using a scenario with M∈{0,1,2,3,4} migration events, we initiated a graph search from a random initial graph with Mbuti set as the outgroup, and the algorithm for 1000 iterations. The graph search was rerun if the optimal graph with *M* migration events did not have a better score than those with fewer events. Comparing score distributions between 1000 bootstrap replicates of *f*_2_ blocks, we found that the best-fit model to have three migration events.

### (k) Statistical analysis

All statistical analyses employed in this work were previously implemented within the scope of the above-mentioned tools and methods.

## Data Availability

All genomes generated for this paper are available via the NCBI SRA, PRJNA883375. The data are provided in electronic supplementary material [37].
